# Hernia, Mesh, and Topical Antibiotics, Especially Gentamycin: Seeking the Evidence for the Perfect Outcome…

**DOI:** 10.3389/fsurg.2014.00053

**Published:** 2015-02-04

**Authors:** Hakan Kulacoglu

**Affiliations:** ^1^Department of Surgery, Recep Tayyip Erdoğan University, Rize, Turkey

**Keywords:** inguinal hernia, mesh, infection, prophylaxis, gentamycin

## Abstract

Inguinal hernia repair is a clean surgical procedure and surgical site infection (SSI) rate is generally below 2%. Antibiotic prophylaxis is not routinely recommended, but it may be a good choice for institutions with high rates of wound infection (>5%). Typical prophylaxis is the intravenous application of first or second-generation cephalosporins before the skin incision. However, SSI rate remains more than 2% in many centers in spite of intravenous antibiotic prophylaxis. Even a 1% SSI rate may be unacceptable for the surgeons who specifically deal with hernia surgery. A hernia center targets to be a center of excellence not only in respect of recurrence rate but also for other postoperative outcomes, therefore a further measure is required for an excellent result regarding infection control. Topical gentamycin application in combination with preoperative single-dose intravenous antibiotic may be a useful to obtain this perfect outcome. Data about this subject are not complete and high-grade evidence has not been cumulated yet. Prospective randomized controlled trials can make our knowledge more solid about this subject and help the surgeons who seek perfect outcome regarding infection control in inguinal hernia surgery.

## Introduction

Surgical site infection (SSI) is an annoying complication for both surgeons and patients. SSI consumes extra time and money, accounting for 20% of all healthcare-associated infections (1). SSI develops in 5% of patients (2). SSIs can increase in-hospital time and repetitive admissions to outpatient clinics. When a prosthetic material is used, an extra problem may arise: necessary extraction of the prosthesis because of a deep and resistant infection.

Inguinal hernia repair is a clean surgical procedure and the SSI rate is generally below 2%; however, every institution has its own infection figure and many of them cannot produce that low rate. General hospitals, especially centers who receive every kind of surgical case, including trauma and contaminated cases, may have SSI rates >5% (3–5). Antibiotic prophylaxis, not routinely recommended for inguinal hernia repair, may be a good choice for institutions with high rates of wound infection (>5%) (6).

Typical antibiotic prophylaxis for an inguinal hernia repair is the intravenous application of first- or second-generation cephalosporins before skin incision (7, 8). It is possible to lower the SSI rate with this prophylaxis; however, SSI rates remain above 2% in many centers despite intravenous antibiotic prophylaxis. On the other hand, even a 1% SSI rate may be unacceptable for surgeons who specifically deal with hernia surgery. A hernia center aims to be a center of excellence, not only in terms of recurrence rate but also for other postoperative outcomes (9); therefore, a further measure is required for excellent infection control.

## Topical Antibiotics in General

Topical antibiotics have been employed for the treatment of infected wounds. Some antimicrobial agents have also been used topically to reduce SSI rates. Topical antibiotic application has many potential advantages over systematic use, though some specific disadvantages also exist (10). The clinical benefits of topical antibiotic prophylaxis have not been extensively documented for general surgical cases.

Commonly used antibiotics for topical prophylaxis are cephalosporins, aminoglycosides, glycopeptides, chloramphenicol, and bacitracin (10); the doses, delivery methods, and pharmacological profiles vary.

The benefits of topical antibiotic prophylaxis with various agents have been documented for multiple surgical disciplines, including ophthalmic, orthopedic, neurological, urological, and general surgeries (11–16); however, the evidence for inguinal hernia repair with mesh is limited, as recent PubMed and Google Scholar searches found only four controlled clinical studies (5, 17–19).

The first clinical study to examine the merit of topical antibiotic prophylaxis in inguinal hernia repairs was published in 1967 (20), carried out in a hernia-specific institution in Canada, Shouldice Hospital, by Ernest Ryan. He compared test group patients who received topical penicillin solution to controls with no antibiotic prophylaxis. The test group developed a 0.15% wound infection rate whereas the control series had a 1.54% infection rate. Ryan was the first believer of the concept that “all significant infections in clean surgical wounds start during the time the wound is open at operation.” He injected penicillin solution deep to the external oblique aponeurosis after it was closed and the remainder was injected on or into the more superficial tissues.

John Nicholls also used an antibiotic solution, made using penicillin powder, into the deep and superficial tissues during the wound closure of inguinal hernia repairs after he read Ryan’s article (21). He did not observe any local or systematic reaction to penicillin, and recorded 3 wound infections out of a total of 136 patients (2.2%). His wound infection rate is higher than Ryan obtained in Shouldice Hospital, possibly because of type of his institution, which was a general hospital in Seychelles. He also mentioned that only one of his three infections appeared to have arisen spontaneously, the other two being attributable to hematoma due to inadequate hemostasis.

Andersen and colleagues published their experience with topical ampicillin application in patients that underwent McVay repair for inguinal hernias in 1980 (17). They delivered 1 g of ampicillin powder into the subfascial layers in the test group and recorded a 3.7% wound infection rate, whereas the wound infection rate was 4.0% in the control group. They concluded that topical ampicillin application had no effect.

Lazorthes et al., in 1992, published their controlled clinical studies on local antibiotic prophylaxis in inguinal hernia repair (18). Patients who received 750 mg of cefamandole directly into the wound with local anesthesia developed no SSI, compared with a 4.3% wound infection rate in patients who were not given local prophylaxis. The costs of the antibiotics used were 10 times less than the cost of the treatment of wound infections in the control group.

## Gentamycin in Hernia Repair with Mesh

As mesh repairs became the procedure of choice in most centers, intravenous antibiotic prophylaxis gained popularity in inguinal hernia repair but topical antibiotic use for SSI prophylaxis did not. In 2004, the journal Hernia published a letter from Professor Maximo Deysine, MD, FACS (1931–2009) entitled, “Post mesh herniorrhaphy infection control: Are we doing all we can?” (22). He wrote that the postherniorrhaphy infection rate remained at unacceptable levels for the last 30 years, whereas orthopedic surgeons, as a group who utilized prosthetic devices judiciously, decreased their infection rate from 10 to 1%. He continued: “A disturbing fact is that 2–4% infection rate is expected and accepted by most surgeons as a normal outcome of a ‘clean’ procedure. This attitude is similar to that existing in the early 1800s when a 98% plus infection rate was considered ‘normal’ for elective surgery. Later on, the discoveries and recommendations by Pasteur and Lister, departing drastically from what was considered customary, originated a new era in infection control. Their capacity for philosophical change is needed today in the field of herniorrhaphy because we accept, without resistance, figures that represent a very large number of infected patients.”

Deysine was the author of the article entitled, “Pathophysiology, prevention, and management of prosthetic infections in hernia surgery” published in the “Groin Hernia Surgery” issue of the classical series of the Surgical Clinics of North America in 1998 (23). Upon starting at a hernia clinic in 1981, he was faced with five almost-consecutive infections in 1982 that prompted the team to utilize prophylactic intravenous antibiotics (1 g first-generation cephalosporin 30 min before surgery). Following an empirical suggestion from Dr. Wesley Alexander, they started to use a wound irrigation solution of 80 mg gentamycin and 250 mg saline as part of a prospective strict surgical protocol. Deysine reported their results in detail in 2006 and mentioned no SSI in 23 years after a strict protocol for prevention of infection, including topical gentamycin use (24).

As a surgeon who has a specific interest in hernia surgery, this was my first real notice regarding topical gentamycin use in hernia repairs with mesh. I kept reading Deysine’s paper and refreshed my knowledge about gentamycin: it is an amino-sugar linked by a glycosidic bond to a central aminocyclitol ring. It causes a concentration-dependent bacterial killing that is independent of the inoculum’s size, by first diffusing passively across the bacterial pores’ outer membrane and then transversing the membrane by an energy-dependent system. Once in the cytoplasm, gentamycin binds the 30S ribosomal subunit, which leads to faulty reading of mRNA codons, i.e., the wrong amino acids are incorporated into bacterial proteins. The gentamycin killing effect is concentration-dependent and is followed by a bacteriostatic effect. Other killing mechanisms, like electrostatic interaction, have been postulated to explain the rapidity of its lethal effect. Although it is most effective against Gram-negative bacteria, gentamycin also has killing activity against staphylococci, including both *S. aureus* and *S. epidermidis*. The empirical choice of a highly concentrated gentamycin solution may explain our results because, at the concentration used, gentamycin would be bactericidal and not bacteriostatic. Most important, gentamycin shows antimicrobial synergy when used in combination with B-Lactams; thus our preoperative intravenous injection of Cefazolin may have contributed to successful bacterial killing whereupon both activity curves are combined. In other words, B-Lactams seem to increase the antimicrobial effect potential of gentamycin.

In fact, the first paper about topical gentamycin prophylaxis in the prosthetic repair of hernias was published by Musella and colleagues in 2001 (19). In this prospective randomized study, absorbable collagen tampons treated with gentamycin were placed in front of polypropylene mesh. The SSI rate was significantly lower in the test group compared to the control group (0.3 vs. 2.0%). The authors stated that collagen causes faster coagulation, so the occurrence of seroma and hematoma that could favor bacterial proliferation may be reduced. Use of topical gentamycin allows use of a concentration that is much higher than its systemic injection. The blood concentration of antibiotic remains low, reducing ototoxicity and nephrotoxicity, while the local drug concentrations are kept for at least 48 h (25); in this way, resistance to antibiotics caused by low drug dosage is avoided.

Further evidence regarding gentamycin use was published by the Aachen group, which has great experience in hernia surgery and meshes. They have first shown that an antibiotic surface modification of polyvinylidene fluoride (PVDF) mesh samples is feasible with gentamycin with no cytotoxicity (26). Afterward, it was presented that a surface modification of PVDF mesh samples using plasma-induced graft polymerization of acrylic acid and supplementation of gentamycin is able to improve collagen type I/III ratio, scar quality, and mesh integration (27). Later, they showed a dose-dependent effect of gentamycin on MMP-2 expression and tissue integration in a transgenic mouse model (28). The reduced MMP-2 protein expression and transcription after mesh coating with 8 μg/mg gentamycin, together with the improved collagen type I/III, hint at advanced tissue integration, even in the long term. The next paper from the same group revealed that gentamycin-supplemented PVDF mesh materials enhance tissue integration due to transcriptionally reduced MMP-2 protein expression (29). Their most recent study compared plain and modified gentamycin-supplemented PVDF meshes and shown that serum concentrations of antibiotic display a peak value 1 h postoperatively and decline within the next day. The total size of the granuloma was significantly smaller in the gentamycin group compared to the control group. Except for a short period of increased expression of CD68 in the gentamycin group after 7 days, no further difference was found in cellular immune response. The collagen type I/III ratio was constant in the two mesh types without significant differences between mesh materials. A significantly decreased foreign body granuloma formation, compared to the pure PVDF mesh group, was found. *In vitro* analysis showed efficient antibiotic effects of the gentamycin supplementation compared to the pure PVDF mesh (30).

Obviously, all five studies from the Aachen group are experimental rat studies, which do not examine the effects of gentamycin application on clinical SSI rates; however, they provide important data about the potential clinical benefits of topical gentamycin use in inguinal hernia repair with prosthetic materials. These findings may be relevant to the best outcome after mesh repair of abdominal wall hernias.

Like other aminoglycosides, gentamycin is not accepted or suitable for a single-agent prophylaxis in surgical procedures (8); however, gentamycin is used in combination with cefazolin or metronidazole in some centers (31, 32). Nevertheless, most reports about intravenous gentamycin prophylaxis are released from developing and underdeveloped countries (32, 33) and/or for pediatric populations (34, 35). A very recent study from Iran that examined the adherence to American Society of Health System Pharmacists Surgical Antibiotic Prophylaxis Guidelines reported that gentamycin is one of the antibiotics most frequently used inappropriately for intravenous SSI prophylaxis (31).

A recent randomized study from Nigeria, carried out in a pediatric population, showed that single-dose intravenous gentamycin prophylaxis can obtain a virtually zero percent SSI rate, whereas the control group developed a 4.8% wound infection rate (Level of Evidence 2B) (35). Finally, in 2009, Praveen and Rohaizak, from a university hospital in Malaysia, reported that locally applied gentamycin (irrigation solution included 160 mg gentamycin diluted in 250 ml saline) is equivalent to intravenous gentamycin (injection solution included 240 mg gentamycin diluted in 10 ml saline) in preventing SSI in inguinal hernia repair (5) (Table 1). However, the infection rate in both groups was almost 7%, which is not acceptable for this era. They call attention to the responsibility of hematoma formation for the development of wound infection, as Nicholls complained about three decades ago (21).

**Table 1:** **A summary of data regarding topical gentamycine for SSI prophylaxis in inguinal hernia repairs**.

Author	Year	Article type	Benefit	Level of evidence
Musella	2001	Prospective randomized clinical study	Yes	1B
Deysine	2004	Personal experience	Yes	5 (Expert opinion)
Junge	2005	Experimental animal study	Yes	–
Deysine	2006	Retrospective clinical study	Yes	4 (Case series)
Praven	2009	Prospective randomized clinical study	Yes	1B

In fact, preventing hematoma formation reduces wound infection rate in many kinds of surgical procedures (36–39). Surgical experience and measures for good hemostasis can lower hematoma and subsequent infection rates, but a proper antibiotic prophylaxis may still be the key for best outcome after elective hernia repairs. As mentioned earlier in this paper, a one or two percent SSI rate can be deemed as acceptable in general, but every surgeon, especially one who has a special interest to one particular field of surgery, really does not like, even hates, facing a wound infection after an elective procedure in his specific interest area. Therefore, as in the aphorism, “the perfect is the enemy of the good,” a perfect antibiotic prophylaxis with intravenous single-dose first-generation cephalosporin and topical gentamycin may be the way to the very best outcome, regarding infection control in elective inguinal hernia repair.

## Summary

Elective inguinal repair is a clean surgical procedure that has a <2% SSI rate. Proper intravenous antibiotic prophylaxis provides that low infection rate in many institutions; however, it is possible to almost eliminate wound infection following elective inguinal repair by using topical gentamycin application in combination with preoperative single-dose intravenous antibiotic. Data about this subject are not complete and high-grade evidence has not been collected yet. Prospective randomized controlled trials can solidify our knowledge about this subject and help surgeons who seek perfect SSI outcomes in inguinal hernia surgery.

## Conflict of Interest Statement

The author declares that the research was conducted in the absence of any commercial or financial relationships that could be construed as a potential conflict of interest.

## References

[1] de Lissovoy G Fraeman K Hutchins V Murphy D Song D Vaughn BB. Surgical site infection: incidence and impact on hospital utilization and treatment costs. Am J Infect Control (2009) 37(5):387–97. 10.1016/j.ajic.2008.12.010 19398246

[2] Gottrup F Prevention of surgical-wound infections. N Engl J Med (2000) 342(3):202–4 10.1056/NEJM200001203420310 10639548

[3] Ergul Z Akinci M Ugurlu C Kulacoglu H Yilmaz KB. Prophylactic antibiotic use in elective inguinal hernioplasty in a trauma center. Hernia (2012) 16(2):145–51. 10.1007/s10029-011-0881-2 21928096

[4] Yerdel MA Akin EB Dolalan S Turkcapar AG Pehlivan M Gecim IE Effect of single-dose prophylactic ampicillin and sulbactam on wound infection after tension-free inguinal hernia repair with polypropylene mesh: the randomized, double-blind, prospective trial. Ann Surg (2001) 233(1):26–33. 10.1097/00000658-200101000-00005 11141221 PMC1421162

[5] Praveen S Rohaizak M. Local antibiotics are equivalent to intravenous antibiotics in the prevention of superficial wound infection in inguinal hernioplasty. Asian J Surg (2009) 32(1):59–63. 10.1016/S1015-9584(09)60011-7 19321405

[6] Miserez M Peeters E Aufenacker T Bouillot JL Campanelli G Conze J Update with level 1 studies of the European hernia society guidelines on the treatment of inguinal hernia in adult patients. Hernia (2014) 18(2):151–63 10.1007/s10029-014-1252-6 24647885

[7] D’Amico DF Parimbelli P Ruffolo C. Antibiotic prophylaxis in clean surgery: breast surgery and hernia repair. J Chemother (2001) 1(1):108–11. 10.1179/joc.2001.13.Supplement-2.108 11936352

[8] Bratzler DW Dellinger EP Olsen KM Perl TM Auwaerter PG Bolon MK Clinical practice guidelines for antimicrobial prophylaxis in surgery. Am J Health Syst Pharm (2013) 70(3):195–283 10.2146/ajhp120568 23327981

[9] Köckerling F Berger D Jost JO. What is a certified hernia center? The example of the German hernia society and German society of general and visceral surgery. Front Surg (2014) 1:26. 10.3389/fsurg.2014.00026 25593950 PMC4287162

[10] McHugh SM Collins CJ Corrigan MA Hill AD Humphreys H. The role of topical antibiotics used as prophylaxis in surgical site infection prevention. J Antimicrob Chemother (2011) 66(4):693–701. 10.1093/jac/dkr009 21393223

[11] Shorstein NH Winthrop KL Herrinton LJ. Decreased postoperative endophthalmitis rate after institution of intracameral antibiotics in a Northern California eye department. J Cataract Refract Surg (2013) 39(1):8–14. 10.1016/j.jcrs.2012.07.031 23036356

[12] Yan H He J Chen S Yu S Fan C. Intrawound application of vancomycin reduces wound infection after open release of post-traumatic stiff elbows: a retrospective comparative study. J Shoulder Elbow Surg (2014) 23(5):686–92. 10.1016/j.jse.2014.01.049 24745317

[13] Tai CC Want S Quraishi NA Batten J Kalra M Hughes SP. Antibiotic prophylaxis in surgery of the intervertebral disc. A comparison between gentamicin and cefuroxime. J Bone Joint Surg Br (2002) 84(7):1036–9. 10.1302/0301-620X.84B7.12862 12358368

[14] O’Connor LT Jr Goldstein M. Topical perioperative antibiotic prophylaxis for minor clean inguinal surgery. J Am Coll Surg (2002) 194(4):407–10. 10.1016/S1072-7515(02)01117-1 11949745

[15] Hoffman E Prophylactic antibiotic use in clean surgical procedures. Am Surg (1984) 50(3):161–4. 6703529

[16] Neri V Fersini A Ambrosi A Tartaglia N Valentino TP. Umbilical port-site complications in laparoscopic cholecystectomy: role of topical antibiotic therapy. JSLS (2008) 12(2):126–32. 18435883 PMC3016169

[17] Andersen JR Burcharth F Larsen HW Røder O Andersen B. Polyglycolic acid, silk, and topical ampicillin. Their use in hernia repair and cholecystectomy. Arch Surg (1980) 115(3):293–5. 10.1001/archsurg.1980.01380030041009 6243916

[18] Lazorthes F Chiotasso P Massip P Materre JP Sarkissian M. Local antibiotic prophylaxis in inguinal hernia repair. Surg Gynecol Obstet (1992) 175(6):569–70. 1448739

[19] Musella M Guido A Musella S. Collagen tampons as aminoglycoside carriers to reduce postoperative infection rate in prosthetic repair of groin hernias. Eur J Surg (2001) 167(2):130–2. 10.1080/110241501750070592 11266253

[20] Ryan EA Wound infection prevention by topical antibiotics. Br J Surg (1967) 54(5):324–9 10.1002/bjs.1800540503 6023086

[21] Nicholls JC. Necessity into choice. An appraisal of inguinal herniorrhaphy under local anaesthesia. Ann R Coll Surg Engl (1977) 59(2):124–7. 843045 PMC2491739

[22] Deysine M Post mesh herniorrhaphy infection control: are we doing all we can? Hernia (2004) 8(2):90–1 10.1007/s10029-003-0176-3 15108053

[23] Deysine M. Pathophysiology, prevention, and management of prosthetic infections in hernia surgery. Surg Clin North Am (1998) 78(6):1105–15,viii. 10.1016/S0039-6109(05)70372-8 9927987

[24] Deysine M. Infection control in a hernia clinic: 24 year results of aseptic and antiseptic measure implementation in 4,620 “clean cases”. Hernia (2006) 10(1):25–9. 10.1007/s10029-005-0028-4 16088357

[25] Moore RD Lietman PS Smith CR. Clinical response to aminoglycoside therapy: importance of the ratio of peak concentration to minimal inhibitory concentration. J Infect Dis (1987) 155(1):93–9. 10.1093/infdis/155.1.93 3540140

[26] Junge K Rosch R Klinge U Krones C Klosterhalfen B Mertens PR Gentamicin supplementation of polyvinylidenfluoride mesh materials for infection prophylaxis. Biomaterials (2005) 26(7):787–93. 10.1016/j.biomaterials.2004.02.070 15350784

[27] Junge K Klinge U Rosch R Lynen P Binnebösel M Conze J Improved collagen type I/III ratio at the interface of gentamicin-supplemented polyvinylidenfluoride mesh materials. Langenbecks Arch Surg (2007) 392(4):465–71. 10.1007/s00423-006-0138-1 17242896

[28] Binnebösel M Ricken C Klink CD Junge K Jansen M Schumpelick V Impact of gentamicin-supplemented polyvinylidenfluoride mesh materials on MMP-2 expression and tissue integration in a transgenic mice model. Langenbecks Arch Surg (2010) 395(4):413–20. 10.1007/s00423-010-0601-x 20155364

[29] Binnebösel M von Trotha KT Ricken C Klink CD Junge K Conze J Gentamicin supplemented polyvinylidenfluoride mesh materials enhance tissue integration due to a transcriptionally reduced MMP-2 protein expression. BMC Surg (2012) 12:1. 10.1186/1471-2482-12-1 22244356 PMC3296653

[30] Klink CD Binnebösel M Lambertz A Alizai HP Roeth A Otto J In vitro and in vivo characteristics of gentamicin-supplemented polyvinylidenfluoride mesh materials. J Biomed Mater Res A (2012) 100(5):1195–202. 10.1002/jbm.a.34066 22344710

[31] Rafati M Shiva A Ahmadi A Habibi O. Adherence to American society of health-system pharmacists surgical antibiotic prophylaxis guidelines in a teaching hospital. J Res Pharm Pract (2014) 3(2):62–6. 10.4103/2279-042X.137075 25114939 PMC4124682

[32] Lyimo FM Massinde AN Kidenya BR Konje ET Mshana SE. Single dose of gentamicin in combination with metronidazole versus multiple doses for prevention of post-caesarean infection at Bugando medical centre in Mwanza, Tanzania: a randomized, equivalence, controlled trial. BMC Pregnancy Childbirth (2013) 13:123. 10.1186/1471-2393-13-123 23721411 PMC3681664

[33] Chen YS Liu YH Kunin CM Huang JK Tsai CC. Use of prophylactic antibiotics in surgery at a medical center in southern Taiwan. J Formos Med Assoc (2002) 101(11):741–8. 12517052

[34] Haessler D Reverdy ME Neidecker J Brûlé P Ninet J Lehot JJ. Antibiotic prophylaxis with cefazolin and gentamicin in cardiac surgery for children less than ten kilograms. J Cardiothorac Vasc Anesth (2003) 17(2):221–5. 10.1053/jcan.2003.51 12698406

[35] Usang UE Sowande OA Adejuyigbe O Bakare TI Ademuyiwa OA. The role of preoperative antibiotics in the prevention of wound infection after day case surgery for inguinal hernia in children in Ile Ife, Nigeria. Pediatr Surg Int (2008) 24(10):1181–5. 10.1007/s00383-008-2241-6 18726104

[36] Fry DE. The prevention of surgical site infection in elective colon surgery. Scientifica (Cairo) (2013) 2013:896297. 10.1155/2013/896297 24455434 PMC3881664

[37] Olsen MA Butler AM Willers DM Devkota P Gross GA Fraser VJ. Risk factors for surgical site infection after low transverse cesarean section. Infect Control Hosp Epidemiol (2008) 29(6):477–84. 10.1086/587810 18510455

[38] Acklin YP Widmer AF Renner RM Frei R Gross T. Unexpectedly increased rate of surgical site infections following implant surgery for hip fractures: problem solution with the bundle approach. Injury (2011) 42(2):209–16. 10.1016/j.injury.2010.09.039 21047637

[39] Vilar-Compte D Rosales S Hernandez-Mello N Maafs E Volkow P. Surveillance, control, and prevention of surgical site infections in breast cancer surgery: a 5-year experience. Am J Infect Control (2009) 37(8):674–9. 10.1016/j.ajic.2009.02.010 19556033

